# Management of Suspected Stage IVA Endometrial Cancer With Rectosigmoid Involvement Using Neoadjuvant Chemotherapy and Interval Surgery

**DOI:** 10.7759/cureus.101986

**Published:** 2026-01-21

**Authors:** Yogeeta Gunasagran, Kenneth Lim

**Affiliations:** 1 Gynaecologic Oncology, Universiti Malaya Medical Centre, Kuala Lumpur, MYS; 2 Gynaecologic Oncology, University Hospital of Wales, Cardiff, GBR

**Keywords:** advanced endometrial cancer, endometrioid endometrial carcinoma, neoadjuvant chemo radiation, postmenopausal bleeding, total laparoscopic hysterectomy (tlh) with bilateral salpingo-oophorectomy

## Abstract

Stage IVA endometrial cancer with rectosigmoid involvement is rare and presents challenges for primary surgical management due to the risk of major multivisceral resection and associated morbidity. We report the case of a 55-year-old woman who presented with prolonged postmenopausal bleeding, foul-smelling vaginal discharge, and pelvic pain. Imaging showed a large pelvic mass inseparable from the rectum and sigmoid colon. Histopathology demonstrated a moderately differentiated adenocarcinoma of gynaecological origin that was mismatch repair-proficient and p53 wild-type. In view of the extent of local disease and the anticipated morbidity of primary surgery, the multidisciplinary team (MDT) recommended neoadjuvant chemotherapy (NACT). The patient experienced rapid symptomatic improvement following the first cycle of NACT. Baseline cross-sectional imaging demonstrated a large pelvic mass measuring 14x10 cm, with suspected rectosigmoid involvement and suspicious pelvic lymphadenopathy. Following four cycles of chemotherapy, repeat imaging showed a marked reduction in tumour size consistent with a partial radiological response according to RECIST 1.1 (Response Evaluation Criteria in Solid Tumours version 1.1) criteria. No progressive nodal disease was identified.

In view of the favourable radiological and clinical response, minimally invasive interval surgery was planned with the primary aim of assessing disease resectability, with the intention to proceed with cytoreductive surgery only in the absence of widespread peritoneal disease. Interval laparoscopic hysterectomy and bilateral salpingo-oophorectomy were subsequently performed. Intraoperatively, dense fibrosis and tissue friability were encountered, consistent with chemotherapy response. There was no macroscopic peritoneal disease, and no evidence of true rectosigmoid invasion; therefore, bowel resection was not required. Final histopathology demonstrated only focal residual adenocarcinoma involving the endometrium and cervix with clear margins. The patient received one further cycle of chemotherapy postoperatively and is currently awaiting adjuvant radiotherapy. She remains symptom-free at follow-up.

This case highlights the role of NACT in selected patients with locally advanced endometrial cancer, where primary surgery carries high morbidity. It demonstrates that endometrioid carcinomas can achieve meaningful clinical and pathological responses, enabling interval surgery in advanced-stage disease through careful MDT-led decision-making.

## Introduction

Endometrial cancer is the fourth most common cancer in women in the United Kingdom and the most common gynaecological malignancy.Incidence has been increasing over recent decades, rising by approximately 59% since the early 1990s and by a further 10% in the last decade [1]. The majority of cases are diagnosed at an early stage, with around 80% presenting with stage I disease and five-year survival exceeding 95%. In contrast, outcomes are substantially poorer in the presence of regional or distant spread, with five-year survival rates of approximately 68% and 17%, respectively [2].

The management of endometrial cancer has historically centred on primary surgical treatment, typically comprising total hysterectomy with bilateral salpingo-oophorectomy, with or without lymph node assessment depending on preoperative risk factors and intraoperative findings. Primary surgery allows accurate FIGO (International Federation of Gynecology and Obstetrics) staging [3] and provides essential pathological information, including tumour grade, depth of myometrial invasion, lymphovascular space involvement (LVSI), and molecular classification, all of which are used for postoperative risk stratification. Adjuvant therapy, including vaginal brachytherapy, external beam radiotherapy, and systemic chemotherapy, is then tailored to the individual’s risk group to reduce recurrence and improve survival. This surgery-first approach is effective for the majority of patients, particularly those diagnosed at an early stage. However, in situations where the tumour is initially unresectable or where multivisceral surgery would entail disproportionate morbidity, systemic therapy may be considered as the first step in management.

Retrospective series and population-level analyses suggest that neoadjuvant chemotherapy (NACT) with platinum and taxane, followed by interval cytoreductive surgery, is increasingly used in advanced endometrial cancer, achieving higher optimal cytoreduction rates, reduced perioperative morbidity, and similar survival outcomes compared with primary cytoreduction alone [4].

Although early NACT experience focused primarily on endometrial serous histologies [5], more recent studies have demonstrated that this strategy may also be effective in high-grade and selected endometrioid carcinomas, particularly when local invasion of adjacent organs makes primary surgery technically challenging or high risk [6]. Nevertheless, reports specifically describing the use of NACT in stage IVA endometrioid endometrial cancer with suspected rectal or rectosigmoid involvement remain limited.

We describe a case of suspected stage IVA endometrioid endometrial carcinoma with MRI-based suspected rectal/rectosigmoid invasion that was successfully managed with neoadjuvant platinum-taxane chemotherapy followed by interval cytoreductive surgery, illustrating the clinical reasoning, multidisciplinary team (MDT) decision-making, and evolving evidence supporting this approach in complex, locally advanced disease.

## Case presentation

A 55-year-old postmenopausal woman, five years since her last menstrual period, presented to her general practitioner (GP) with a several-month history of worsening foul-smelling, fluid-like vaginal discharge. This was preceded by intermittent vaginal bleeding over a two-year period. She also reported progressively worsening dull lower abdominal pain requiring regular oral analgesia, with increasing frequency in recent months. Over the preceding six months, she noted an unintentional weight loss of 6 kg accompanied by reduced appetite, and her BMI was 24.5 kg/m². According to her history, she had one vaginal delivery 15 years prior. Her last cervical cytology at the age of 46 was normal but overdue for repeat screening. There was no significant family history of cancer. She smoked approximately eight cigarettes per day.

In view of her symptoms, she was referred by her general practitioner to the Urgent Suspected Cancer Clinic. While awaiting gynaecological assessment, she was empirically treated for presumed pelvic infection with oral antibiotics. Initial abdominal examination revealed a palpable pelvic mass consistent with a 14-week-sized uterus. There were no palpable inguinal or pelvic lymph nodes. On vaginal examination, a craggy, irregular mass occupied the upper half of the vagina, and the cervix could not be identified separately from the lesion. With an initial clinical suspicion of cervical malignancy, a punch biopsy of the vaginal mass was performed.

**Figure 1:**
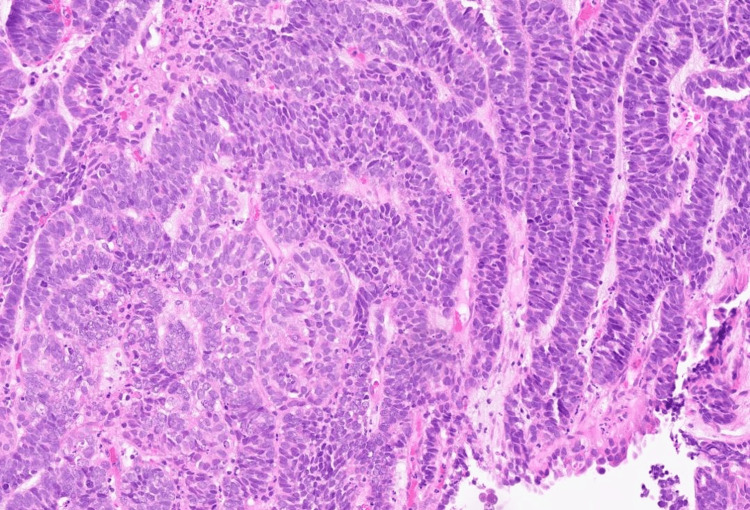
Photomicrograph of the cervical biopsy representing endometrioid adenocarcinoma

Histopathology demonstrated a moderately differentiated adenocarcinoma with necrosis of gynaecological tract origin (Figure 1). Possible primary sites included the endometrium or endocervix. Immunohistochemistry showed tumour cells were PAX8 positive, focally CK7 positive, and very focally carcinoembryonic antigen (CEA) positive. They were negative for CK20, CDX2, p63, p16, vimentin, and oestrogen receptor (Allred score 0/8). The tumour exhibited wild-type p53 staining and retained expression of all mismatch repair (MMR) proteins (MLH1, PMS2, MSH2, and MSH6), consistent with MMR-proficient status.

**Figure 2:**
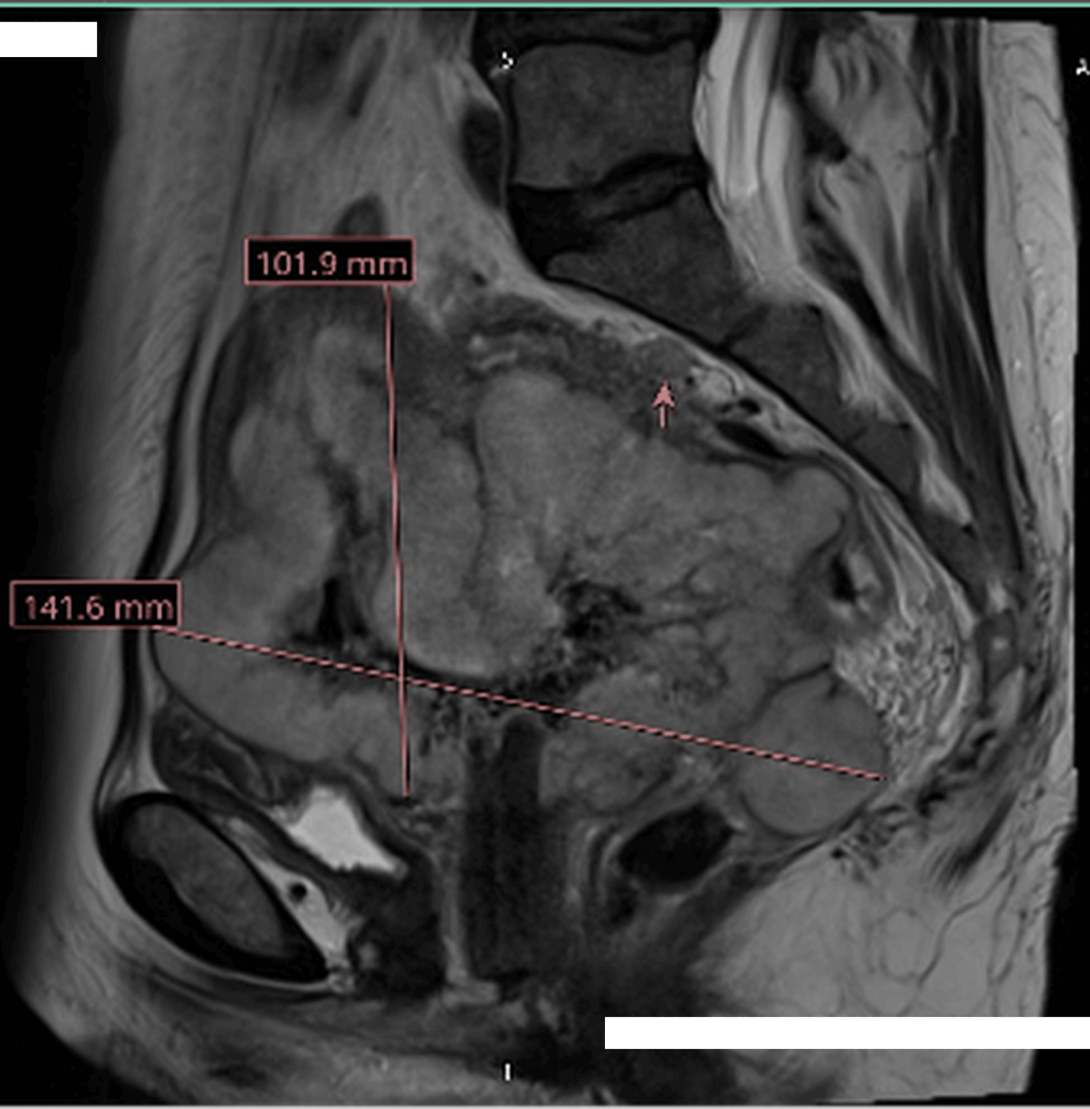
MRI pelvis showing a 10.1 cm x14.1 cm pelvic mass. The pink arrow is pointing towards a suspicious right obturator lymph node.

**Figure 3:**
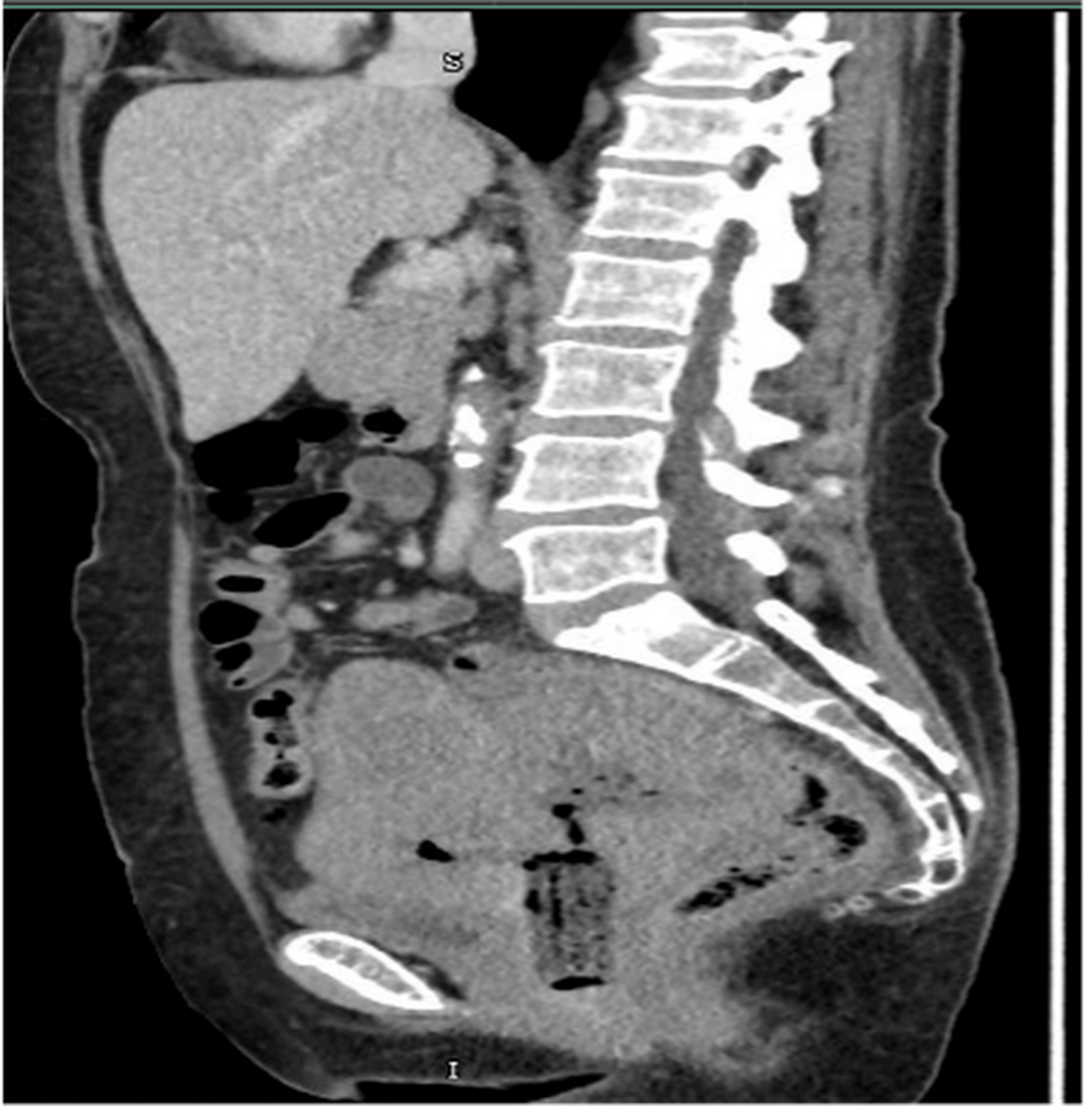
CT (sagittal view) showing a huge pelvic mass that is inseparable from the bladder and rectosigmoid.

Following review of the histology, MRI of the pelvis (Figure 2) and CT of the thorax, abdomen, and pelvis (Figure 3) were arranged, which revealed a 14 × 10 cm lobulated, T2 intermediate signal mass replacing the uterus and cervix. The tumour extended into the parametria bilaterally and abutted the anterior bladder wall without definite mucosal invasion. Posteriorly, the mass was inseparable from the rectum and sigmoid colon, though there was no convincing evidence of bowel mucosal invasion. Suspicious lymph nodes were identified in the right obturator and para-aortic regions. No hydroureteronephrosis was noted. CT imaging confirmed the pelvic findings and showed no distant metastatic disease.

**Figure 4:**
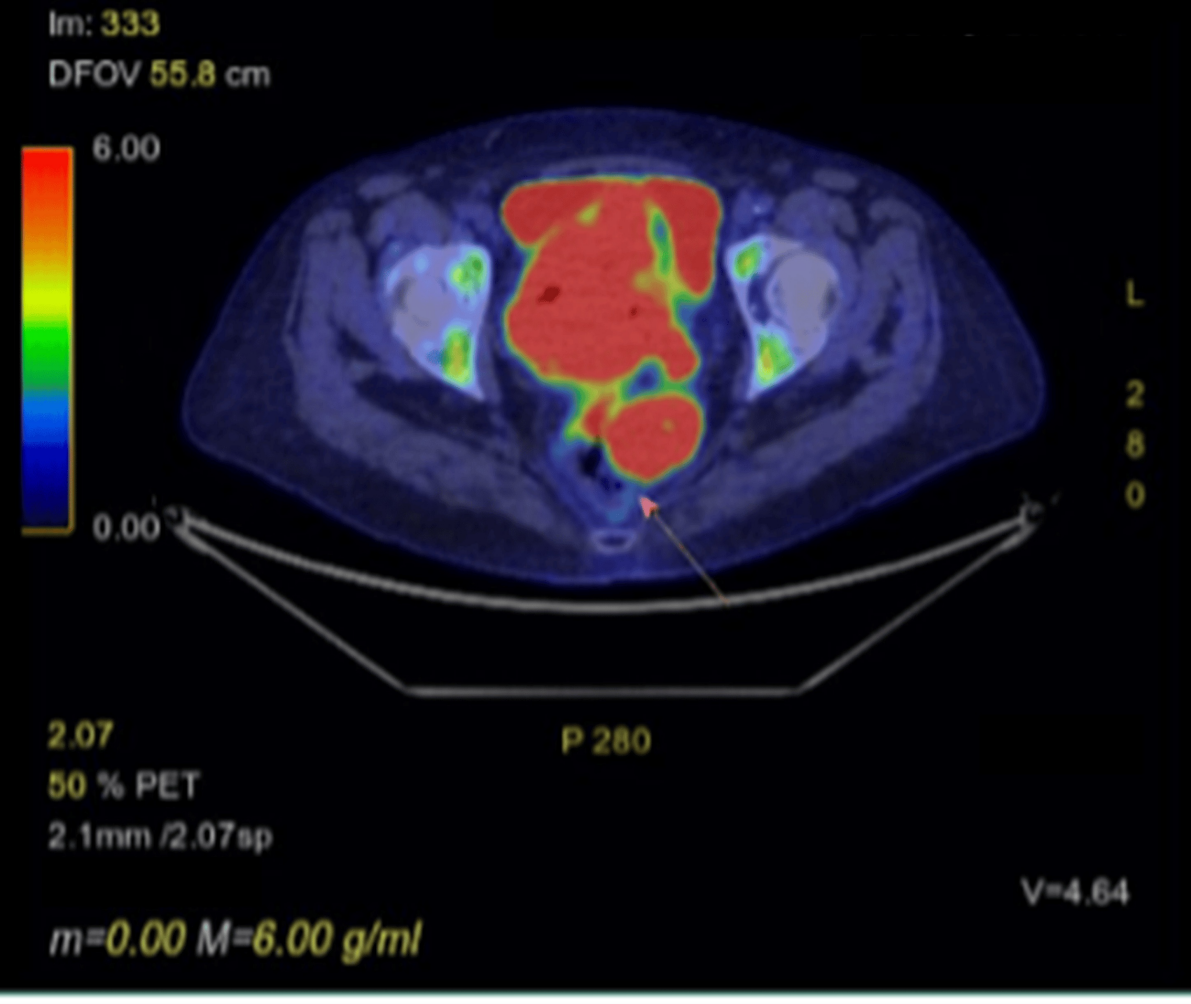
PET scan showing involvement of rectum (arrow)

**Figure 5:**
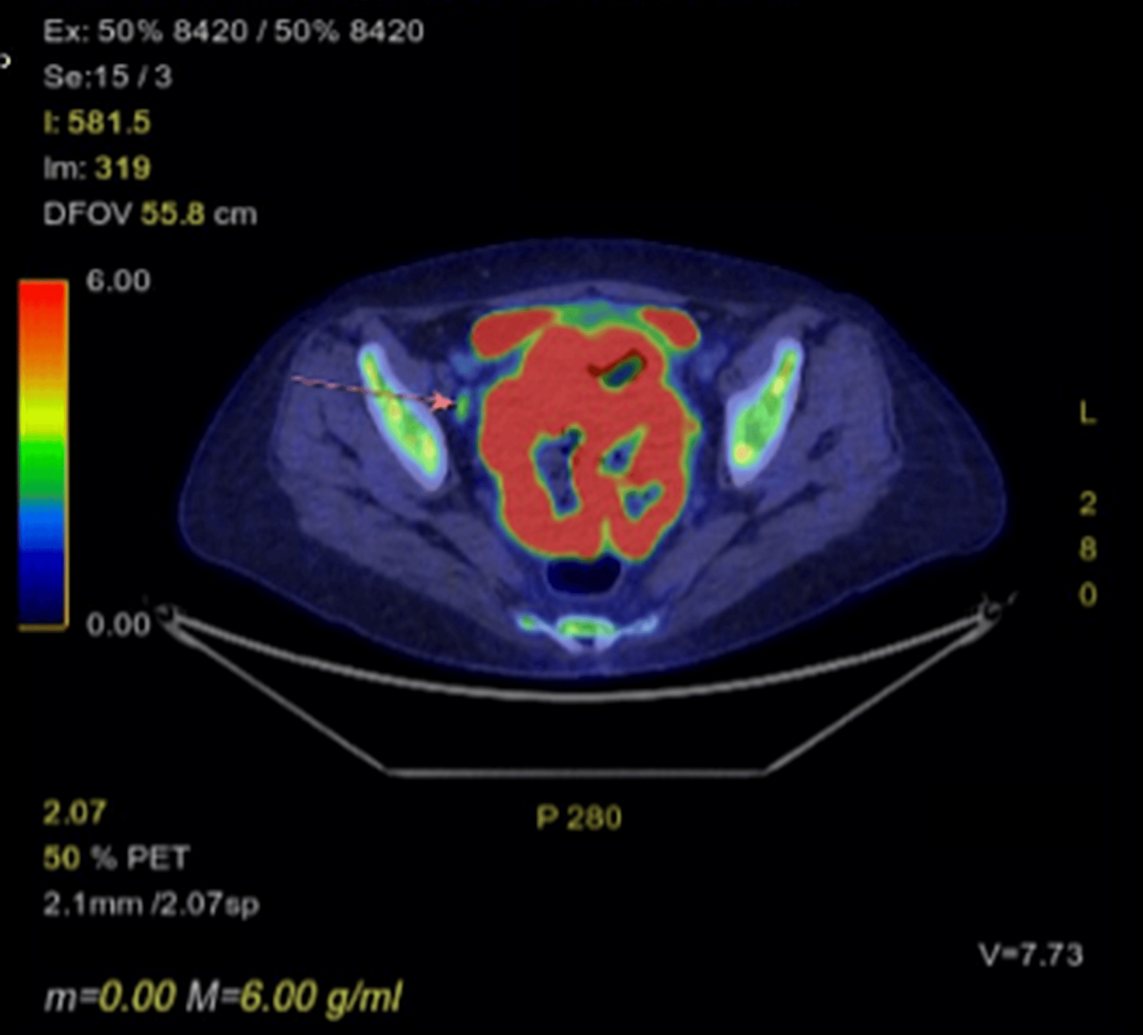
PET scan showing enlarged right external iliac lymph node (arrow)

A PET-CT scan was performed approximately two weeks after the MRI and CT. This demonstrated a large, predominantly necrotic but extremely fluorodeoxyglucose (FDG)-avid pelvic malignancy extending into the left mesorectal space. Cross-sectional imaging demonstrated loss of the normal fat plane between the pelvic mass and the rectum, with appearances suggesting involvement of the rectum at this site (Figure 4). Two mildly FDG-avid lymph nodes were noted: one adjacent to the right external iliac vein (Figure 5) and one within the left para-aortic region. Both demonstrated only low-grade uptake, discordant with the intense FDG avidity of the primary tumour, and were therefore considered potentially reactive rather than metastatic.

**Figure 6:**
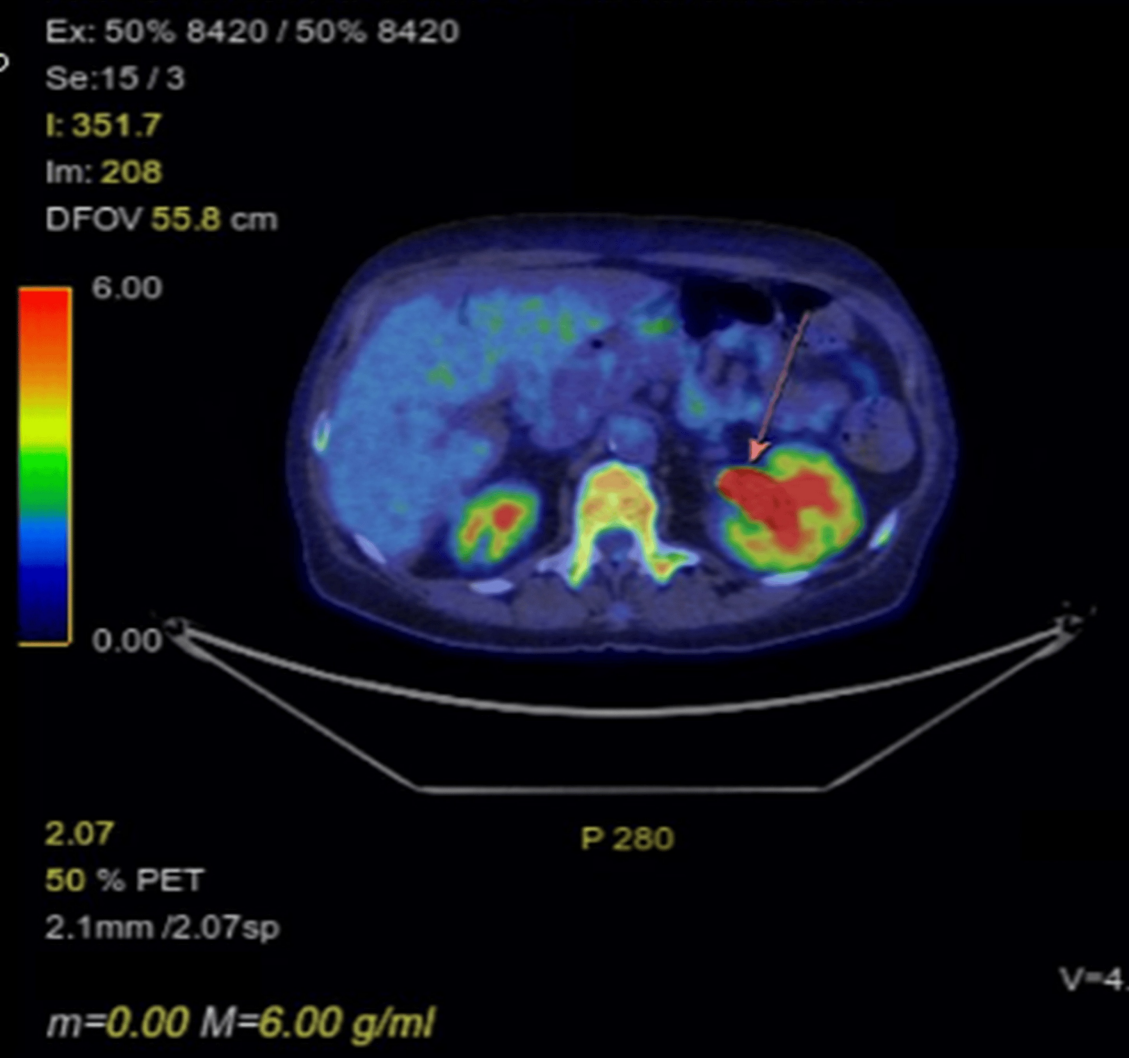
PET-CT showing left hydroureteronephrosis (arrow)

The pelvic tumour had progressed sufficiently to cause moderate left-sided hydronephrosis and hydroureter (Figure 6); however, renal function was still preserved. There was no biochemical evidence of obstructive uropathy, and the patient remained clinically stable without features of sepsis or acute kidney injury. In this context, urgent urinary diversion was not required, and it was considered safe to proceed with systemic chemotherapy with close renal monitoring. No FDG-avid distant metastatic disease was identified. Endoscopic assessment was considered; however, given the absence of luminal obstruction on imaging, the consensus of the multidisciplinary team (MDT) was not to proceed with endoscopic evaluation at that stage. 

Following review of all imaging at the MDT meeting, a provisional diagnosis of stage IVA endometrial carcinoma was made. The MDT concluded that primary surgery would carry substantial morbidity given the extent of local disease. It was therefore agreed that NACT would be the most appropriate initial management.

The patient proceeded to receive four cycles of carboplatin and a taxane. Her first two cycles included paclitaxel; however, following a grade 3 hypersensitivity reaction, paclitaxel was switched to nab-paclitaxel (Abraxane). After the first cycle of chemotherapy, the patient reported marked improvement in her foul-smelling vaginal discharge and a reduction in analgesic requirements. She was also reviewed by the dietitian and commenced on high-protein nutritional supplements.

**Figure 7:**
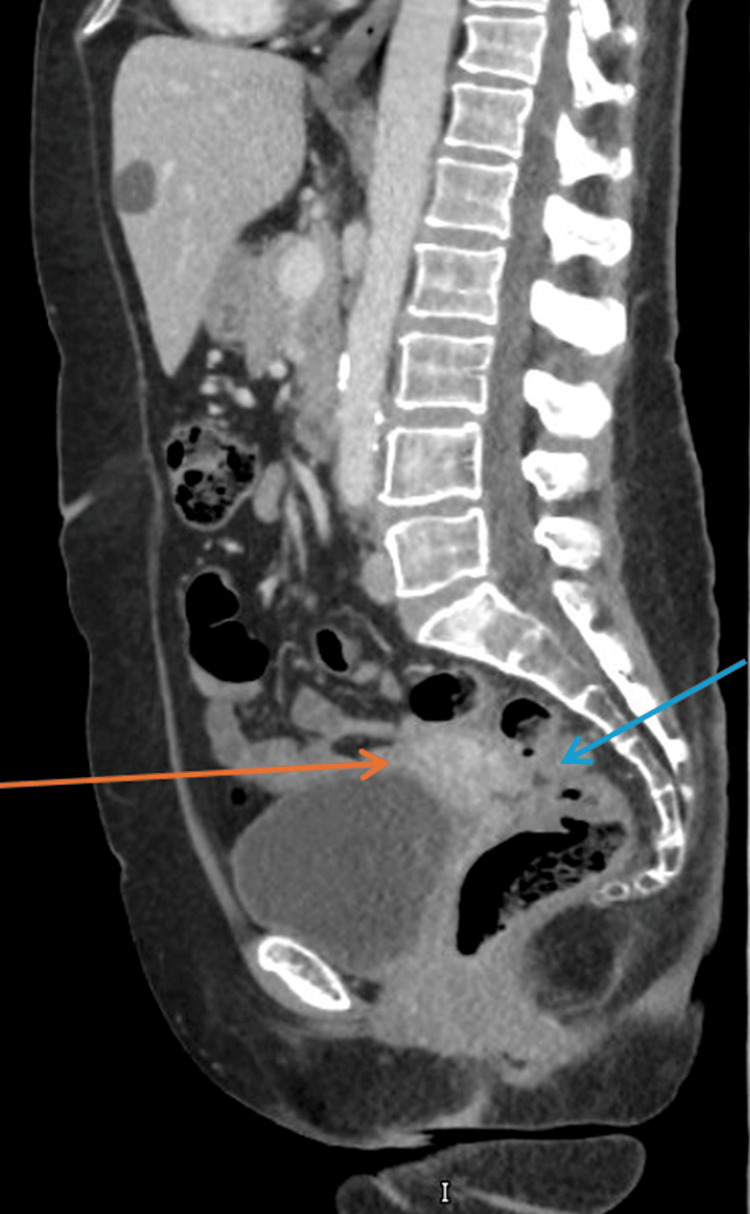
CT of thorax, abdomen and pelvis after four cycles of neoadjuvant chemotherapy, showing uterus has shrunk (orange arrow) and rectosigmoid appears to be inseparable from the uterus (blue arrow)

A repeat CT of the thorax, abdomen, and pelvis was performed after four cycles of NACT (Figure 7). This demonstrated an excellent response, with a substantial reduction in the volume of the primary endometrial tumour. However, the pelvic small bowel and rectum/sigmoid colon remained inseparable from the uterus, suggesting persistent dense adherence or possible residual tumour involvement.

Given the excellent radiological and symptomatic response to NACT, the MDT recommended that interval cytoreductive surgery could now be considered. The plan was to proceed with a hysterectomy and bilateral salpingo-oophorectomy to achieve removal of the primary tumour, with a view to postoperative radiotherapy if no macroscopic peritoneal disease was identified intraoperatively.

**Figure 8:**
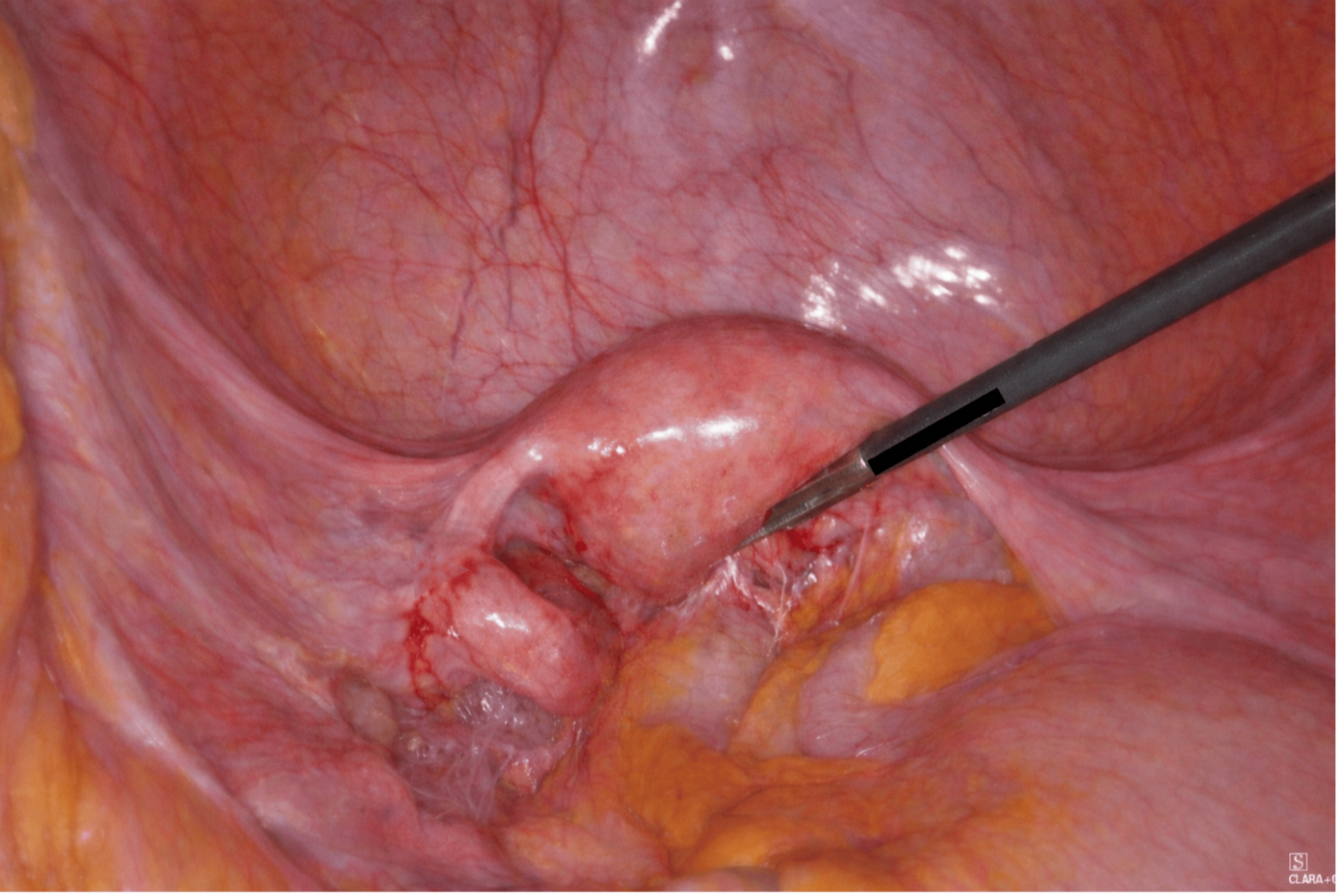
Intraoperative photo showing adhesions of the rectum and adnexa to the posterior wall of uterus obscuring the Pouch of Douglas.

The patient subsequently underwent a laparoscopic hysterectomy and bilateral salpingo-oophorectomy. Intraoperatively, cervical stenosis prevented the passage of a uterine manipulator. The uterus was not enlarged. Dense adhesions involving the sigmoid colon and adnexae required careful blunt and sharp adhesiolysis (Figure 8). The bladder was firmly adherent to the cervix. During the dissection of the uterine arteries, the cervix was inadvertently separated from the uterine body, reflecting the friable nature of the tissues. No obvious peritoneal disease was seen. Formal nodal assessment was not undertaken at the time of surgery. This decision was multifactorial, reflecting extensive post-NACT fibrosis, operative complexity, and the anticipated need for adjuvant radiotherapy irrespective of nodal status. In this context, the additional operative risk associated with nodal dissection or sentinel lymph node mapping was felt to outweigh its potential staging benefit. The patient made an uncomplicated recovery and was discharged on postoperative day one.

According to the final histopathology report, the uterus and cervix appeared almost normal grossly. Microscopically, there were small foci of residual adenocarcinoma involving the endometrium and cervix. No definite myometrial invasion was identified. The ovaries and fallopian tubes were free of tumour. The cervix showed no background cervical glandular intraepithelial neoplasia (CGIN), and p16 remained negative. As with the initial biopsy, the tumour was MMR-proficient and demonstrated wild-type p53 staining. The tumour appeared clear of cervical resection margins.

Following surgery, the patient received an additional cycle of carboplatin and nab-paclitaxel as consolidation chemotherapy. She is currently awaiting a final decision and scheduling for adjuvant radiotherapy. At the time of this case report, the patient had completed two months of postoperative follow-up. During this period, she remained clinically well, asymptomatic, and without evidence of disease recurrence on clinical assessment. She is currently having chemotherapy as part of adjuvant treatment.

## Discussion

Suspected stage IVA endometrial cancer with rectal or rectosigmoid involvement represents a particularly challenging clinical scenario. In such presentations, the extent of local disease often precludes safe primary surgery, and attempts at en bloc resection may necessitate extensive bowel surgery associated with substantial morbidity. In this patient, cross-sectional imaging demonstrated a large pelvic tumour inseparable from the rectosigmoid colon, raising significant concern regarding the feasibility of achieving complete cytoreduction at the outset.

Although pre-treatment imaging suggested rectal involvement and possible FIGO stage IVA disease, mucosal invasion was not definitively demonstrated, and bowel resection was ultimately not required at surgery. It is therefore plausible that this case represents advanced locally invasive endometrial carcinoma rather than confirmed stage IVA disease with true mucosal involvement. Nevertheless, NACT served an important clinical purpose by reducing tumour bulk and local inflammatory reaction, thereby improving operability and allowing definitive surgery without the need for multivisceral resection. This highlights the potential role of NACT as a strategy to facilitate organ preservation in selected patients with extensive pelvic disease where the extent of invasion is uncertain.

While the initial diagnostic biopsy was obtained from a vaginal mass in the context of a clinically unidentifiable cervix, the primary site was ultimately established as endometrial. Pelvic MRI demonstrated a dominant uterine mass with extension into the cervix and upper vagina, consistent with downward spread from an endometrial primary. Definitive histopathological assessment of the hysterectomy specimen confirmed an endometrial carcinoma with secondary cervical involvement. There was no evidence of cervical glandular intraepithelial neoplasia or an alternative primary cervical malignancy, supporting the classification of this tumour as endometrial in origin.

Neoadjuvant platinum-taxane chemotherapy was therefore selected following MDT discussion. The decision was based not only on the predicted morbidity of primary surgery but also on emerging evidence demonstrating that neoadjuvant treatment can downstage disease sufficiently to enable interval cytoreduction in advanced endometrial cancer. Currently, the European Society of Gynaecological Oncology (ESGO)-European Society for Radiotherapy and Oncology (ESTRO)-European Society of Pathology (ESP) guidelines acknowledge the role of systemic therapy as initial treatment in unresectable or medically inoperable advanced disease, with surgery considered once adequate tumour regression has been obtained [7]. Importantly, several studies have shown that patients who respond to neoadjuvant therapy and subsequently undergo interval surgery achieve survival outcomes comparable to those treated with primary surgery, with the added benefit of lower perioperative morbidity [8]. This appears to hold true even in endometrioid tumours, which historically were not the main focus of early NACT cohorts.

Clinical response to NACT was assessed using symptomatic, nutritional, and radiological parameters. Prior to treatment, the patient reported progressive pelvic pain and vaginal bleeding requiring regular opioid analgesia. Following initiation of chemotherapy, there was a marked improvement in pain control, allowing stepwise de-escalation of analgesic requirements, along with resolution of abnormal vaginal bleeding. Nutritional status improved, with weight stabilisation and improved oral intake during chemotherapy. Serial imaging demonstrated interval improvement consistent with a reduction in pelvic tumour burden.

Intraoperative findings of tissue friability and dense fibrosis are consistent with treatment response and have been well described in patients receiving neoadjuvant therapy [4,6].The near-normal gross appearance of the uterus and cervix, coupled with only focal microscopic residual adenocarcinoma and absence of myometrial invasion, supports a strong pathological response to chemotherapy, an observation that adds to the growing body of evidence that selected endometrioid carcinomas can be chemo-responsive [6,7].

Another important consideration is the potential prognostic benefit of interval debulking surgery. Several studies have reported that patients who receive NACT but do not proceed to surgery have significantly poorer outcomes, whereas those who achieve optimal cytoreduction experience improved progression-free and overall survival [4,6]. In this case, interval laparoscopic hysterectomy and bilateral salpingo-oophorectomy could be performed safely, with no evidence of peritoneal disease, supporting the notion that downstaging through NACT can create an opportunity for meaningful surgical cytoreduction in advanced presentations.

Molecular profiling has increasing relevance in endometrial cancer, although its clinical utility is most established in early-stage disease, where polymerase ε (POLE)-ultramutated tumours may allow adjuvant de-escalation [8]. In advanced-stage presentations such as this, POLE testing is not routinely performed as it does not influence initial treatment decisions. This tumour demonstrated MMR proficiency and wild-type p53 staining, features commonly seen in endometrioid carcinomas. Although estrogen receptor (ER) negativity is atypical for endometrioid histology, the marked radiological and pathological response observed in this case is consistent with reports that selected endometrioid tumours can be chemoresponsive [6,8].

This case contributes to the relatively limited literature describing NACT in stage IVA endometrioid carcinoma with suspected bowel involvement. It highlights the importance of individualised decision-making, the value of MDT assessment, and the potential role of NACT in expanding surgical options for patients in whom primary surgery carries an unacceptably high risk. As evidence for this approach grows, it may become an increasingly relevant strategy in selected patients with locally advanced endometrial cancer. Prospective data collection and larger cohort studies are needed to better define patient selection, optimise treatment sequencing, and clarify oncological outcomes for patients with advanced-stage endometrial cancer managed with NACT and interval cytoreduction.

## Conclusions

Suspected stage IVA endometrioid endometrial cancer with rectosigmoid involvement is uncommon, and primary cytoreductive surgery in this setting may be associated with significant morbidity. In selected patients, NACT can achieve meaningful clinical and radiological downstaging, leading to symptom improvement and enabling interval hysterectomy in those initially unsuitable for upfront surgery. However, radiological response does not always translate into technically easier surgery, as treatment-related fibrosis and tissue friability may still be encountered intraoperatively.

Patients who respond to NACT and subsequently undergo interval debulking appear to have improved outcomes compared with those managed with chemotherapy alone, supporting reassessment for surgical intervention following treatment. While molecular features such as MMR proficiency and p53 wild-type status may assist prognostic stratification, management decisions in advanced disease continue to rely heavily on radiological assessment, MDT discussion, and careful consideration of anticipated surgical morbidity.
